# Supplementary material for: Identification of photoperiod-induced specific miRNAs in the adrenal glands of Sunite sheep (Ovis aries)
Source: Front Vet Sci. 2022 Jul 22;9:888207. doi: 10.3389/fvets.2022.888207 (PMC9354845; doi:10.3389/fvets.2022.888207)
Supplement: Supplementary file 1 [file Data_Sheet_1.ZIP › Supplementary file/miRDeep2 file.zip!/miRDeep2 file/Know_Structure/SP42A3/oar-mir-376a.pdf]

miRBase precursor : oar-mir-376a
Total read count : 83
oar-miR-376a-5p read count : 49
oar-miR-376a-3p read count : 32
remaining reads : 2

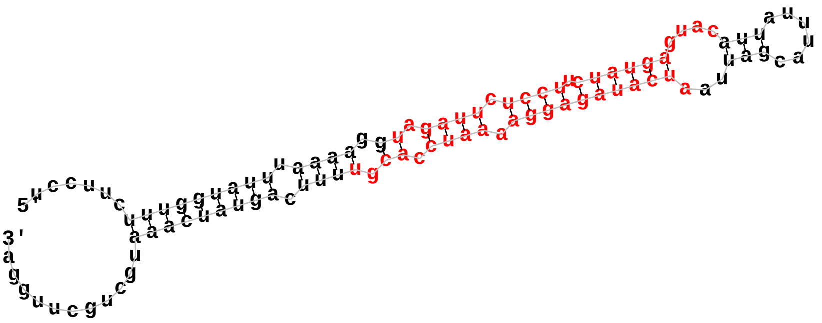

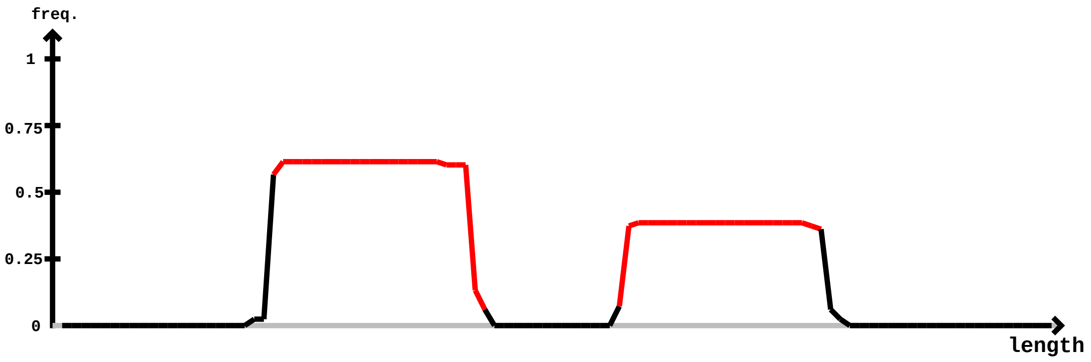

oar-miR-376a-5p

oar-miR-376a-3p

5'- uccuucuuugguauuuaaaagguagauucuccuucuaugaguacauuauuuacgauuaaucauagaggaaaauccacguuuucaguaucaaaugcugcuugga -3' exp

......((((((((.((((.((.((((.((((.((((((....(((......)))...)))))))))).)))).)).)))).))))))))..............	reads	mm	sample
...................agguagauucuccuucuaugagu...	2	0	seq
.....................guagauucuccuucuaug..	1	0	seq
.....................guagauucuccuucuaugagu...	35	0	seq
.....................guagauucuccuucuaugagua..	5	0	seq
.....................guagauucuccuucuaugaguac...	4	0	seq
......................uagauucuccuucuaugagu...	2	0	seq
......................uagauucuccuucuaugagua..	1	0	seq
......................uagauucuccuucuaugaguac...	1	0	seq
....aaucauagaggaaaauccacgu........................	5	0	seq
....aaucauagaggaaaauccacguu.......................	1	0	seq
....aucauagaggaaaauccac..........................	1	0	seq
....aucauagaggaaaauccacg.........................	1	0	seq
....aucauagaggaaaauccacgu........................	20	0	seq
....aucauagaggaaaauccacguu.......................	2	0	seq
....aucauagaggaaaauccacguuu......................	1	0	seq
....ucauagaggaaaauccacguuu......................	1	0	seq
